# Coffee, Samba, Football and… Social Inequalities: Reflections on Mortality in São Paulo, Brazil

**DOI:** 10.1590/S1516-31802001000300001

**Published:** 2001-05-02

The most important newspapers and magazines worldwide (The New York Times, Le Monde, The Wall Street Journal, Time magazine) have been publishing the tremendous results from the Brazilian AIDS Program. This is a combination of preventive measures and free distribution of antiretroviral drugs with an impressive fall in hospitalization and mortality. Now, the Brazilian program has become the paramount public health effort for halting the AIDS epidemic to be followed in other places, especially the sub-Saharan African countries. In contrast, the Health Department of São Paulo State is warning about increasing case-fatality rates of tuberculosis. How can this contradiction be explained for a non-Brazilian observer?

For us, Brazilian physicians and medical researchers, the answer is very easy. AIDS is a disease that has afflicted people with real prestige in Brazilian circles of power. In summary: journalists, popular music stars, soap-opera actors and actress, physicians, scientists, military officers, priests and other well-born citizens who traveled to the USA during the early 1980's and were infected from sexual intercourse in New York City and San Francisco. After that, they spread the virus and disease among Brazilians who had never dreamed of going up the steps into an airplane. Until the early 1990's, in São Paulo City, the incidence of AIDS cases continued to be greater among affluent people than among poor ones. As the spread of AIDS became a menace to the abovementioned professions, it was easy to pressure the Ministry of Health, State Departments of Health, and other authorities to increase the budget for AIDS control and treatment, including sometimes a shift of resources from other programs. The results have been marvelous, but the questions have still not been answered.

The analysis of mortality patterns in São Paulo, made by Marcos Drummond and Marilisa A. Barros,^1^ provides an important contribution in this respect. They divided the districts of Sao Paulo into four homogenous areas in accordance with a socioeconomic status (SES) stratification that is very popular among demographers and social scientists. They studied the death rates in the city in 1991-92 among people aged 15 to 64 years old. Figure 1 shows the death rates according to age strata for men (a), and women (b), respectively. It can be observed that the gap between residents of the richest and the poorest areas is greater among women than among men. The sub-analyses by groups of causes can explain these disparities.

**Figure 1:**
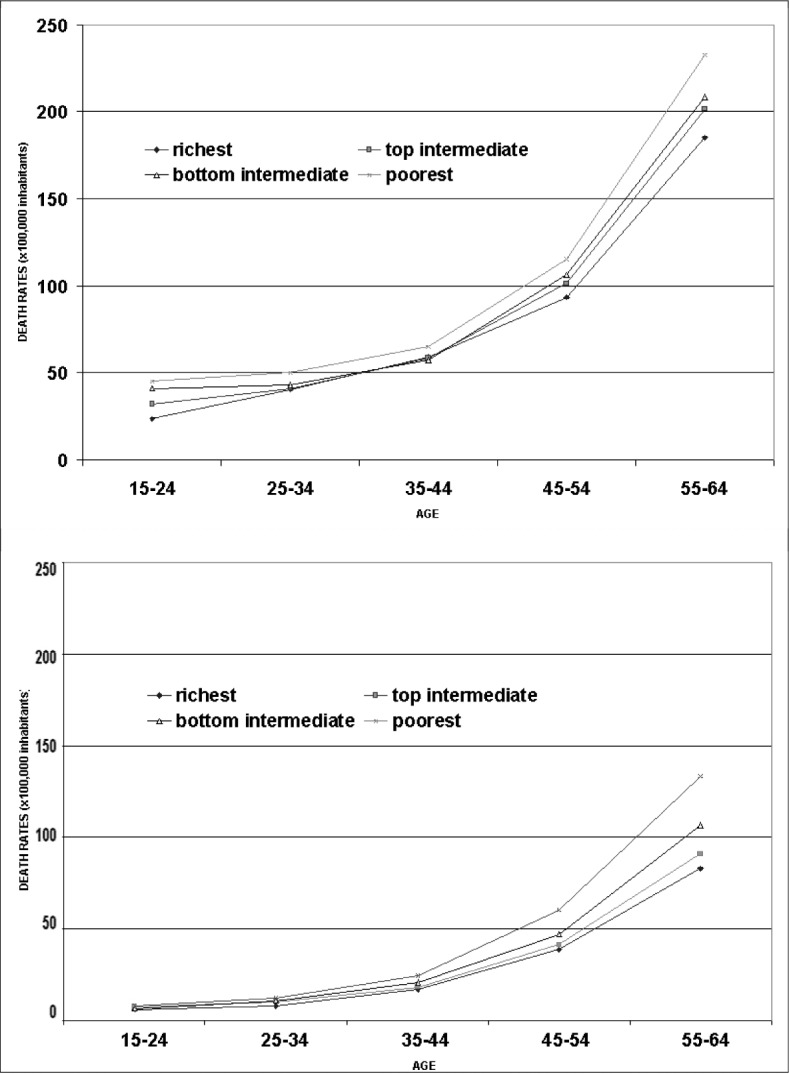
Age-adjusted death rates among men (top) and women (bottom) according to residency area classified by socioeconomic status (Drummond,1999).

Table 1 shows the main causes of death among men, in which statistical significance between death rates was found when comparing the rates obtained in the extreme SES areas. It is obvious that for men, only AIDS mortality was more frequent among inhabitants of the richest area when compared to other areas. Homicide, stroke, alcohol-related disorders and heart failure had a higher toll among poorer people. Table 2 shows the causes of death with statistical significance among women. AIDS was not exposed because no differences were detected. However, the relative risks were 1.0, 0.95, 0.89 and 0.75 from the richest to the poorest area. Stroke, myocardial infarction, pneumonia, diabetes, hypertension, heart failure, Chagas’ disease and tuberculosis were more common as causes of death among poor people.

**Table 1:** Mortality rates among men in the richest homogenous area of São Paulo city and age-adjusted relative risk for death in the other three areas using the richest one as reference. Only diseases whose relative risk comparing the poorest to the richest area reached statistical significance are presented

	Richest	Top intermediate	Bottom intermediate	Poorest
	Reference area, given relative risk = 1*	Relative risk	Relative risk	Relative risk
AIDS	110.3	0.65	0.51	0.33
Homicide	55.4	1.58	1.71	2.95
Stroke	22.5	1.51	1.86	2.34
Pneumonia	20.7	1.39	1.45	1.50
Cirrhosis	19.6	1.43	1.41	1.48
Heart failure	4.9	1.62	1.76	2.15
Chagas' disease	2.9	1.15	1.90	3.23
Hypertension	5.1	1.77	2.08	3.02
Drowning	3.0	1.82	2.83	3.78

* *Rate (×100,000); Source: Drummond M, Barros MBA. Rev Bras Epidemiol 1999;2(1/2):34-49.*

**Table 2:** Mortality rates among women in the richest homogenous area of São Paulo city and age-adjusted relative risk for death in the other three areas using the richest one as reference. Only diseases whose relative risk comparing the poorest to the richest area reached statistical significance are presented

	Richest	Top intermediate	Bottom intermediate	Poorest
	Reference area, given relative risk = 1*	Relative risk	Relative risk	Relative risk
Stroke	18.0	1.07	1.55	2.26
Myocardial Infarction	13.4	1.35	1.56	2.02
Pneumonia	7.8	1.08	1.36	1.80
Diabetes	4.4	1.54	2.32	2.83
Hypertension	4.1	1.42	1.70	2.87
Heart failure	2.9	1.52	2.06	2.74
Chagas' disease	1.2	1.50	2.64	4.27
Tuberculosis	1.1	1.92	2.78	3.51

*Source: Drummond M, Barros MBA. Rev Bras Epidemiol 1999;2:34-49.*

The uniform pattern observed for men and women reveals some important (and bitter) aspects of the utilization of public resources in Brazil. The richest have had a revolutionary and radical public health program that has left most public heath lawmakers astonished. In contrast, the poorest have not had medical care capable of avoiding the bad effects of living in neighborhoods lacking the minimum decent living conditions, with high criminality, bad schools, and with many of the hospital and health center facilities having few full-time doctors and nursing staff.

It seems incredible, but some Brazilian public heath authorities were until recently still proclaiming that cardiovascular diseases were a group of diseases that only affected rich people in a significant way. Municipalities governed by left-wing parties were not exempt from the belief that cardiovascular disease need not be considered as a priority in closing the gap between death rates of different social groups. In addition, São Paulo City was governed from 1993 to 2000 by a populist right-wing regime that destroyed the epidemiological surveillance system, in the same way that another popu-list, Mayor Giuliani, did in New York City with the tuberculosis program.^2^

Thus, it is possible to understand why Brazilian solves some problems that are considered very hard to control by public health authorities elsewhere, such as the AIDS epidemic. However, we cannot control high blood pressure and tuberculosis among the poorer people. We know that social inequality is universal and has been a constant from Biblical times until our times, but the capability for shifting public resources from the poorer to the richer is something that has as much of a Brazilian taste as coffee, samba, and football.

## References

[1] Drummond M, Barros MBA (1999). Social Inequalities in Adult Mortality in São Paulo City. Rev Bras Epidemiol.

[2] Garrett L (1996). The coming plague. Newly emerging diseases in a world out of balance.

